# Juvenile dermatomyositis: clinical and laboratory charateristics of 18 patients

**DOI:** 10.1186/1546-0096-9-S1-P50

**Published:** 2011-09-14

**Authors:** E Iglesias, J Antón, S Ricart, J Ros, V Torrente, R Bou, MA González, A Vicente

**Affiliations:** 1 Department of Pediatric Infectious Diseases and Immunodeficiencies, Hospital Infantil Virgen del Rocío, Seville, Spain; 2 Pediatric Rheumatology Unit; 3 Department of Dermatology, Hospital Sant Joan de Deu, Esplugues de Llobregat, Barcelona, Spain

## Introduction

Juvenile Dermatomyositis (JDM) is the most common idiopathic inflammatory myopathy in childhood, a systemic vasculopathy that affects usually skin, skeletal muscle, gastrointestinal tract and other organs. The diagnosis is made by criteria and the goals of treatment include control the underlying myositis and prevent disease and treatment complications. Stepwise aggressive treatment decrease long term sequelae and improve outcomes with a more rapid normalization of muscle inflammation.

## Methods

We studied demographics, epidemiology, clinical and laboratory characteristics in a descriptive observational study of 18 children with JDM assisted at our hospital from January 2000 to November 2010.

## Results

72.2% of patients were females. Mean age at disease onset was 7.36 years. Most common presenting features were cutaneous and weakness. Medium time between first signs/symptoms and diagnosis was 59 days. 81.2% had pathological CK at diagnosis. CMAS was pathological in 85.7% of patients, electromyography in 84.61% and magnetic resonance image (MRI) in all patients that was made. Muscular biopsy was compatible with JDM in 83.33%. Medium time between first symptoms and treatment beginning was 1.94 months. Drugs more frequently used were corticosteorids, methotrexate, hidroxicloroquine and intravenosus immunoglobulines. Time to response was 7.12 months. 50% of patients had disease relapse (66.6% of them with normal muscular enzymes) and medium time for it was 6.63 months. Actually, 7/16 patients have reached clinical remission, 6/16 complete clinical response, 2/16 clinical response.

## Conclusions

1. Other myositis-enzymes than CK should be tested at diagnosis.

2. MRI could evaluate muscular edema with a no invasive technique.

3. Muscular enzymes are a good tool to monitor disease activity but in some patients wonÂ´t be useful, so we have to consider MRI and other activity markers.

